# Sarcopenia and Sarcopenic Obesity as Novel Risk Factors for Gastric Carcinogenesis: A Health Checkup Cohort Study

**DOI:** 10.3389/fonc.2019.01249

**Published:** 2019-11-14

**Authors:** Young Min Kim, Jie-Hyun Kim, Su Jung Baik, Jaeyoung Chun, Young Hoon Youn, Hyojin Park

**Affiliations:** ^1^Department of Internal Medicine, Gangnam Severance Hospital, Yonsei University College of Medicine, Seoul, South Korea; ^2^Department of Healthcare Research Team, Health Promotion Center, Gangnam Severance Hospital, Yonsei University College of Medicine, Seoul, South Korea

**Keywords:** gastric carcinogenesis, sarcopenia, sarcopenic obesity, metabolic syndrome, risk factor

## Abstract

**Background:** Insulin resistance, the primary mechanism of metabolic syndrome, promotes gastric carcinogenesis. Metabolic syndrome is associated with sarcopenia. We aimed to investigate the association between sarcopenia and gastric carcinogenesis, including precancerous conditions such as atrophic gastritis (AG), intestinal metaplasia (IM), and dysplasia.

**Methods:** The study included adult patients who underwent gastroduodenoscopy at a checkup center. AG and IM were evaluated using endoscopy. Based on muscle mass, sarcopenia was defined as a skeletal muscle index <1 standard deviation below the sex-specific mean for healthy adults aged 20–39 years (cutoff point: 29.3% for males and 26.7% for females). Obesity was defined as a body mass index (BMI) ≥25 kg/m^2^ according to the Asia-Pacific criteria. Sarcopenic obesity was defined as a combination of sarcopenia and obesity. The association between gastric carcinogenesis and sarcopenia was evaluated.

**Results:** Among 8,356 enrolled participants, 0.14 and 42.5% were diagnosed with gastric cancer and precancerous conditions, respectively. Approximately 41.7% of gastric cancer patients and 16.9% of patients with precancerous conditions were diagnosed with sarcopenia. Both sarcopenic obesity (odds ratio [OR] = 4.139, *P* = 0.016) and diabetes mellitus (DM) (OR = 5.152, *P* = 0.005) were significantly associated with gastric cancer. Sarcopenia, DM, hypertension, dyslipidemia, *Helicobacter pylori* infection, smoking, and alcohol consumption were significantly associated with precancerous conditions.

**Conclusions:** Sarcopenia and sarcopenic obesity were associated with gastric carcinogenesis and may be novel risk factors for gastric carcinogenesis.

## Introduction

Gastric cancer is one of the commonest malignancies, especially in East Asian countries, such as Korea and Japan (1). Although people aged ≥40 years in Korea undergo biannual endoscopy as part of the National Cancer Screening program, Korea remains one of the countries with the highest incidence of gastric cancer in the world (2). For this reason, it is important to not only detect precancerous conditions or early gastric cancer through screening endoscopy but also control modifiable risk factors. Gastric cancer is a multistep and multifactorial disease. Precancerous conditions, including atrophic gastritis (AG), intestinal metaplasia (IM), and dysplasia, progress to gastric cancer because of multiple influencing factors (3, 4). To date, several risk factors such as *Helicobacter pylori* infection, diabetes mellitus (DM), obesity, smoking, alcohol consumption, and diet, including the consumption of salty or smoked foods, are known to be associated with gastric carcinogenesis (5, 6). Among these factors, DM and obesity are components of metabolic syndrome. Insulin resistance, the primary mechanism of metabolic syndrome, promotes the carcinogenesis of various malignancies, including gastric cancer (7).

Sarcopenia is defined as the progressive loss of muscle mass and function as a result of the aging process. It has been recognized as a major health problem because of its association with the risk of adverse outcomes, such as physical disability, poor quality of life, and death (8). With the aging of population, the prevalence of sarcopenia has been increasing, although it can vary according to the definition, method used to measure muscle mass, age, race, and sex (9, 10). When the BIA method was used, the prevalence in the non-Asian population was higher than that in the Asian population for men (19 vs. 10%), and the prevalence in the non-Asian population was higher than that in the Asian population for women (20 vs. 11%). When dual-energy X-ray absorptiometry (DXA) was used, the prevalence in the Asian population was higher than that in the non-Asian population for men (9 vs. 6%), and the prevalence in the non-Asian was higher than that in the Asian population for women (10 vs. 6%). Previous studies reported that sarcopenia is associated with metabolic syndrome. As skeletal muscle is the primary tissue responsible for insulin-dependent glucose uptake, sarcopenia is associated with systemic insulin resistance, which progressively induces metabolic syndrome (11, 12). Recently, a “new concept,” sarcopenic obesity, which refers to the coexistence of sarcopenia and obesity and which are both associated with metabolic syndrome, has a greater effect on metabolic disease, morbidity, and mortality than sarcopenia and obesity individually (13).

Because both gastric carcinogenesis and sarcopenia are associated with metabolic syndrome, we hypothesized that there is an association between gastric carcinogenesis and sarcopenia. To the best of our knowledge, only a limited number of studies have demonstrated this relationship. Hence, this study aimed to investigate risk factors for gastric carcinogenesis with a focus on sarcopenia and sarcopenic obesity among patients who underwent routine health checkups.

## Materials and Methods

### Study Design and Population

This single-center study was conducted at Gangnam Severance Hospital, Seoul, Korea from August 2017 to August 2018. Starting in August 2017, our checkup center could measure skeletal muscle index (SMI). Data were retrospectively collected from electronic medical records of patients who visited our center for a routine health checkup. Overall, 9,332 Korean patients who underwent upper endoscopy at our checkup center were considered for this study. We excluded 976 patients with a history of gastric cancer or any type of cancer (*n* = 869); a history of gastric surgery, including resection of the stomach or gastrectomy (*n* = 65); and incomplete electronic medical records (*n* = 42). As a result, 8,356 patients were eligible and were enrolled in this study.

The study protocol conformed to the ethical guidelines of the World Medical Association Declaration of Helsinki and was approved by the Gangnam Severance Hospital Institutional Review Board (IRB No. 3-2018-0349). Informed consent was not required because this study was a retrospective analysis of existing administrative and clinical data.

### Anthropometric Measurements

Anthropometric parameters of all enrolled patients were measured. Body weight and height were measured to the nearest 0.1 kg and 0.1 cm, respectively, with the patients wearing light-weight clothing without shoes. To measure skeletal muscle mass, we used multi-frequency bioelectrical impedance analysis (ACCUNIQ BC 720; SELVAS healthcare, Korea), which is considered an appropriate alternative to DXA (14). Appendicular skeletal muscle mass (ASM) was calculated as the sum of appendicular lean mass from both the arms and legs (15). BMI was calculated as body weight divided by height squared (kg/m^2^).

We used the definition of sarcopenia developed by Janssen et al. (16); they adopted SMI to establish the prevalence of sarcopenia and defined sarcopenia as an SMI <1 standard deviation below the sex-specific mean for healthy adults aged 20–39 years. SMI was calculated as ASM/body weight × 100 (%). In our study, the cutoff value for sarcopenia was 29.3% for males and 26.7% for females. We defined obesity as BMI ≥25 kg/m^2^ according to the Asia-Pacific criteria (17). Sarcopenic obesity was defined as the coexistence of sarcopenia and obesity.

### Questionnaire

All patients who visited our checkup center were required to complete a questionnaire. The questionnaire included questions on combined DM, hypertension, dyslipidemia, smoking history, alcohol consumption, and diet (consumption of salty and spicy foods). Regarding diet, patients were asked to select “yes” if they consumed salty and spicy foods six times within 7 days.

### Endoscopic Evaluation of the Stomach

All enrolled patients underwent endoscopic examinations using an endoscope (GIF-H260; Olympus Medical Systems, Tokyo, Japan) equipped with an electronic endoscopy system (EVIS LUCERA; Olympus Medical Systems). *H. pylori* infection was detected using the following methods: rapid urease test (CLO test; Delta West, Bentley, Australia), pathology (Giemsa staining), and immunoglobulin G specific for *H. pylor*i in the serum (enzyme-linked fluorescence assay, Vidas [bioMerieux Vitek, Inc. Hazelwood, MO, USA]). If the result of at least one of these three tests was positive, the patient was diagnosed with *H. pylori* infection. Gastric cancer and gastric dysplasia were confirmed by histologic examination of the tissue obtained during endoscopic biopsy. AG and IM were diagnosed based on the gross endoscopic findings reported by endoscopists with at least 3 years of experience.

### Statistical Analysis

Continuous variables were reported as mean ± standard deviation and were compared between groups using the *t*-test or Wilcoxon rank-sum test. Categorical variables were reported as numbers and percentages, and the chi-square test or Fisher's exact test was used to compare categorical variables.

Pearson's correlation analysis was used to evaluate the correlation between two continuous variables. We performed multivariate Cox regression analysis to identify the risk factors affecting gastric cancer and precancerous conditions. We performed two multivariate analyses because sarcopenic obesity is a combination of sarcopenia and obesity. Statistical analysis was performed using SPSS version 23.0 (IBM Corp., Armonk, NY, USA). A two-tailed *P*-value of <0.05 was considered significant.

## Results

### Baseline Characteristics

Table 1 shows the baseline characteristics of the study population. Of 8,356 patients, 4,670 (55.9%) were men, and the mean age was 49.1 ± 11.6 years. The average BMI was 23.9 ± 3.3 kg/m^2^. The mean SMI was 32.2 ± 2.7% in men and 28.7 ± 3.0% in women. Approximately 15.2% of patients were diagnosed with sarcopenia and 13.5% were diagnosed with sarcopenic obesity. Male subjects had a higher prevalence of sarcopenia (18.8% male vs. 10.6% female) and sarcopenic obesity (17.3% men vs. 8.7% women). Approximately 11.2% of patients had DM, 14.3% had dyslipidemia, and 18.7% had hypertension. Overall, 12 patients had gastric cancer. Moreover, 3,622 patients were diagnosed with gastric precancerous conditions. Among these patients, 2,205 had AG, 1,407 had IM, and 10 had gastric dysplasia.

**Table 1:** Baseline characteristics of the study population.

**Characteristics**	**All patients (*N* = 8,356)**
Age (years, mean ± SD)	49.1 ± 11.6
Male (*n*, %)	4,670 (55.9)
Height (cm, mean ± SD)	166.5 ± 8.5
Weight (kg, mean ± SD)	66.5 ± 12.8
BMI (kg/m^2^, mean ± SD)	23.9 ± 3.3
Obesity (*n*, %)	2,839 (34.0)
SMI (%, mean ± SD)	
Male	32.2 ± 2.7
Female	28.7 ± 3.0
Current smoker (*n*, %)	1,479 (17.1)
Alcohol history (*n*, %)	5,593 (66.9)
DM (*n*, %)	940 (11.2)
Dyslipidemia (*n*, %)	1,192 (14.3)
Hypertension (*n*, %)	1,563 (18.7)
Sarcopenia (*n*, %)	1,270 (15.2)
Male	878/4,670 (18.8)
Female	392/3,686 (10.6)
Sarcopenic obesity (*n*, %)	1,129 (13.5)
Male	810/4,670 (17.3)
Female	319/3,686 (8.7)
Diet, salty and/or spicy (*n*, %)	3,398 (40.7)
*H. pylori* infection (*n*, %)	1,217 (14.6)
Gastric cancer (*n*, %)	12 (0.14)
Gastric precancerous conditions (*n*, %)	3,622 (43.3)
Atrophic gastritis (*n*, %)	2,205 (26.4)
Intestinal metaplasia (*n*, %)	1,407 (16.8)
Dysplasia (*n*, %)	10 (0.12)

*BMI, body mass index; SMI, skeletal muscle index; DM, diabetes mellitus; H. pylori, Helicobacter pylori*.

### Risk Factors for Gastric Cancer

Tables 2, 3 show the risk factors for gastric cancer. In the univariate analysis, advanced age, obesity, sarcopenia, sarcopenic obesity, and DM were significantly associated with an increased risk of gastric cancer (Table 2). In the multivariate analyses, sarcopenic obesity (odds ratio [OR] = 4.139, 95% confidence interval [CI]: 1.304–13.133, *P* = 0.016) and DM (OR = 5.152, 95% CI: 1.624–16.347, *P* = 0.005) were significant risk factors for gastric cancer (Table 3).

**Table 2:** Univariate analysis of the risk factors for gastric cancer.

	**Univariate analysis**		
	**Control (*n* = 8,344)**	**Cancer (*n* = 12)**	***P*-value**
Age (years, mean ± SD)	49.0 ± 11.6	58.8± 13.8	0.003
Male (*n*, %)	4,633 (55.9)	7 (58.3)	0.864
Obesity (*n*, %)	2,831 (33.9)	8 (66.7)	0.028
Current smoker (*n*, %)	1,477 (17.7)	2 (16.7)	1.000
Alcohol history (*n*, %)	5,588 (67.0)	5 (41.7)	<0.001
DM (n, %)	935 (11.2)	5 (41.7)	0.007
Dyslipidemia (*n*, %)	1,190 (14.3)	2 (16.7)	0.685
Hypertension (*n*, %)	1,158 (18.7)	5 (41.7)	0.056
Diet, salty and/or spicy (*n*, %)	3,391 (40.6)	7 (58.3)	0.247
*H. pylori* infection (*n*, %)	1,213 (14.5)	4 (33.3)	0.084
Sarcopenia (*n*, %)	1,265 (15.2)	5 (41.7)	0.025
Sarcopenic obesity (*n*, %)	1,124 (13.5)	5 (41.7)	0.016

*DM, diabetes mellitus; H. pylori, Helicobacter pylori*.

**Table 3:** Multivariate analyses of the risk factors for gastric cancer.

	**Multivariate analysis 1**		**Multivariate analysis 2**	
	**OR (95% CI)**	***P*-value**	**OR (95% CI)**	***P*-value**
DM	5.000 (1.575–15.870)	0.006	5.152 (1.624–16.347)	0.005
Sarcopenia	2.122 (0.557–8.086)	0.270	–	–
Obesity	2.522 (0.623–10.203)	0.195	–	–
Sarcopenic obesity	–	–	4.139 (1.304–13.133)	0.016

*OR, odds ratio; DM, diabetes mellitus; CI, confidence interval*.

*Multivariate analysis 1: Adjusted for DM, sarcopenia, and obesity. Multivariate analysis 2: Adjusted for DM and sarcopenic obesity*.

### Risk Factors for Gastric Precancerous Conditions

Tables 4, 5 show the risk factors for gastric precancerous conditions. In the univariate analysis, advanced age, obesity, smoking, alcohol consumption, sarcopenia, sarcopenic obesity, *H. pylori* infection, DM, dyslipidemia, hypertension, and diet, which included consumption of salty and/or spicy foods, were significantly associated with gastric precancerous conditions (Table 4). Among these factors, sarcopenia (OR = 1.201, 95% CI: 1.039–1.387, *P* = 0.013) was independently associated with gastric precancerous conditions. Moreover, smoking (OR = 1.195, 95% CI: 1.081–1.320, *P* = 0.001), alcohol consumption (OR = 1.316, 95% CI: 1.189–1.456, *P* < 0.001), DM (OR = 1.533, 95% CI: 1.320–1.782, *P* < 0.001), hypertension (OR = 1.412, 95% CI: 1.244–1.603, *P* < 0.001), dyslipidemia (OR = 1.552, 95% CI: 1.357–1.775, *P* < 0.001), and *H. pylori* infection (OR = 3.954, 95% CI: 3.454–4.057, *P* < 0.001) were independent risk factors for gastric precancerous conditions (Table 5).

**Table 4:** Univariate analysis of the risk factors for gastric precancerous conditions.

	**Univariate analysis**		
	**Control (*n* = 4,722)**	**Precancerous conditions (*n* = 3,622)**	***P*-value**
Age (years, mean ± SD)	46.0 ± 11.0	53.2 ± 11.0	<0.001
Male (*n*, %)	2,663 (55.2)	2,000 (56.8)	0.156
Obesity (*n*, %)	1,591 (33.0)	1,240 (35.2)	0.035
Current smoker (*n*, %)	1,848 (38.3)	1,475 (41.9)	0.001
Alcohol history (*n*, %)	1,487 (30.8)	1,269 (36.0)	<0.001
DM (n, %)	407 (8.4)	528 (15.0)	<0.001
Dyslipidemia (*n*, %)	556 (11.5)	634 (18.0)	<0.001
Hypertension (*n*, %)	737 (15.3)	821 (23.3)	<0.001
Diet, salty and/or spicy (*n*, %)	1,903 (39.5)	1,488 (42.2)	0.011
*H. pylori* infection (*n*, %)	375 (7.8)	838 (23.8)	<0.001
Sarcopenia (*n*, %)	671 (13.9)	594 (16.9)	<0.001
Sarcopenic obesity (*n*, %)	610 (12.7)	514 (14.6)	0.010

*DM, diabetes mellitus; H. pylori, Helicobacter pylori*.

**Table 5:** Multivariate analyses of the risk factors for gastric precancerous conditions.

	**Multivariate analysis 1**		**Multivariate analysis 2**	
	**OR (95% CI)**	***P*-value**	**OR (95% CI)**	***P*-value**
Current smoker	1.195 (1.081–1.320)	0.001	1.186 (1.075–1.308)	0.001
Alcohol	1.316 (1.189–1.456)	<0.001	1.322 (1.195–1.463)	<0.001
DM	1.533 (1.320–1.782)	<0.001	1.536 (1.322–1.748)	<0.001
Hypertension	1.412 (1.244–1.603)	<0.001	1.411 (1.243–1.601)	<0.001
Dyslipidemia	1.552 (1.357–1.775)	<0.001	1.554 (1.359–1.777)	<0.001
*H. pylori* infection	3.954 (3.454–4.507)	<0.001	3.938 (3.448–4.498)	<0.001
Diet, salty and/or spicy	1.100 (1.003–1.206)	0.054	1.099 (1.003–1.206)	0.054
Sarcopenia	1.201 (1.039–1.387)	0.013	–	–
Obesity	1.934 (0.834–1.045)	0.233	–	–
Sarcopenic obesity	–	–	1.075 (0.942–1.228)	0.284

*OR, odds ratio; DM, diabetes mellitus; H. pylori, Helicobacter pylori; CI, confidence interval*.

*Multivariate analysis 1: Adjusted for smoking, alcohol, DM, hypertension, dyslipidemia, H. pylori infection, diet, sarcopenia, and obesity. Multivariate analysis 2: Adjusted for smoking, alcohol, DM, hypertension, dyslipidemia, H. pylori infection, diet, and sarcopenic obesity*.

## Discussion

Our study showed that sarcopenia and sarcopenic obesity were significantly associated with gastric carcinogenesis. A possible explanation for these associations might be related to metabolic syndrome. DM, hypertension, and dyslipidemia, which are components of metabolic syndrome, are also significantly associated with gastric carcinogenesis in our study.

As reported in previous studies, DM is considered a risk factor for gastric cancer (18–21). There are several suggested mechanisms to explain the pathogenic role of DM in gastric carcinogenesis. Patients with hyperglycemia may develop insulin resistance, and an increase in insulin levels may induce cell proliferation. This process may cause changes in the gastric mucosa and genetic alterations, eventually leading to gastric carcinogenesis (22). Increases in reactive oxygen species caused by DM and high glucose levels are associated with mutational changes in oncogenes and tumor suppressor genes. This change contributes to gastric carcinogenesis (23, 24). The increase in insulin-like growth factors in DM patients plays an important role in the initiation, progression, and metastasis of gastric cancer (25).

In our study, *H. pylori* infection is significantly associated with precancerous conditions. *H. pylori* infection is a well-known risk factor for gastric carcinogenesis as reported in previous studies. Moreover, one prospective study reported that DM and *H. pylori* infection have synergistic effects on gastric carcinogenesis (26). DM may increase the risk of gastric carcinogenesis by stimulating epithelial cell proliferation, which is the initial step in the cascade of gastric carcinogenesis, because of the presence of *H. pylori*. *H. pylori* infection stimulates pancreatic insulin release by increasing gastrin secretion and decreasing the serum somatostatin level (27, 28). One clinical study reported that patients with *H. pylori* infection have higher insulin resistance (29).

Meanwhile, the association between hypertension and gastric cancer is unclear. However, a previous study explained that gastric cancer and hypertension have a common biochemical pathway. Increased levels of inositol triphosphate and cytosolic calcium are involved in the pathogenesis of hypertension and carcinogenesis stage (30).

Previous studies reported the association between obesity and gastric carcinogenesis. A clinical study showed that the effect of obesity on gastric cancer may differ depending on sex (31). In males, obesity was associated with an increased risk of early gastric cancer and well or moderately differentiated adenocarcinoma. In females, obesity was associated with gastric dysplasia regardless of *H. pylori* infection. One meta-analysis conducted in a cohort and case-control study reported that obesity was associated with the risk of gastric cancer in male and non-Asian patients (32). This study showed that both overweight and obesity were associated with the risk of gastric cardia cancer. In our study, obesity was not significantly associated with an increased risk of gastric carcinogenesis. This finding may be due to the fact that stratified analysis was not performed according to sex and the location of the cancer. However, sarcopenic obesity, combined with sarcopenia and obesity, is a risk factor for gastric cancer. Recent studies have shown that sarcopenic obesity affects the development of cardiometabolic diseases (33–37). These studies also reported that sarcopenic obesity has a strong relationship with metabolic syndrome and is associated with a higher risk of metabolic disorders and mortality than obesity or sarcopenia alone.

In 1998, Baumgartner et al. first defined sarcopenia as ASM/height^2^ (kg/m^2^) <2 standard deviations below the mean of a young reference group (38). Although several different definitions of sarcopenia have since been suggested (38, 39), a widely accepted definition suitable for use in research and clinical practice is still pending because of a lack of consensus (8, 13). Similar to gastric carcinogenesis, age-related sarcopenia is also associated with the development of metabolic syndrome. The expression of glucose transporter type 4, which facilitates the uptake of glucose, is related to the volume of skeletal muscle fiber (40). Therefore, sarcopenia can cause insulin intolerance that leads to metabolic syndrome. Sarcopenia and metabolic syndrome develop a vicious cycle (41). The loss of muscle mass because of sarcopenia results in decreased physical activity, causing visceral fat to accumulate in the body. Because skeletal muscle is an insulin-response target tissue, sarcopenia patients develop progressive metabolic syndrome.

Adipose tissue secretes proinflammatory cytokines such as tumor necrosis factor α and interleukin-6, thereby regulating carbohydrate and fat metabolism. These cytokines are also associated with protein degradation; thus, sarcopenia can be accelerated in individuals with long-standing obesity. Therefore, especially in the elderly population, obesity and sarcopenia should also be considered as sarcopenic obesity (42).

Our study has several strengths over previous studies. First, our data were collected from a health checkup cohort. Most health checkup centers routinely obtain patients' anthropometric measurements, including weight, height, and skeletal muscle mass. Not only obesity but also sarcopenia and sarcopenic obesity can be diagnosed through this routine measurement. However, most physicians focus on obesity. Based on our study, this routine measurement may be a useful tool for predicting gastric carcinogenesis. Second, our study analyzed risk factors for gastric carcinogenesis, including gastric cancer and precancerous conditions. Gastric cancer is one of the heterogeneous cancers that involves a multistep process from precancerous conditions to cancer. Therefore, the result of our study indicates the importance of controlling the risk factors of gastric carcinogenesis.

Our study also had several limitations. First, because our data were obtained from an asymptomatic and healthy checkup cohort, only a few patients diagnosed with gastric cancer who underwent screening endoscopy were enrolled in this study. Therefore, a large-scale study is warranted to confirm our results. Second, patient information, such as medical history, alcohol consumption, smoking, and diet, was collected using a questionnaire. However, the questionnaire did not include the exact amount of alcohol consumption, frequency of smoking, and amount and concentration of food components consumed. Third, although current definitions, such as those of the European Working Group on Sarcopenia in Older People, the European Society for Clinical Nutrition and Metabolism Special Interest Groups, and the International Working Group on Sarcopenia, define sarcopenia as muscle mass and function (8, 43–45), our study adopted only muscle mass. This was because our checkup center did not have a device to measure muscle function. Most checkup centers' routine programs only include muscle mass. Therefore, the result of our study may be sufficient to predict gastric carcinogenesis in healthy subjects. Fourth, precancerous conditions, including AG and IM, were diagnosed only based on gross endoscopic findings rather than on biopsy. However, the diagnosis of precancerous conditions was considered reliable as the endoscopists had sufficient experience (at least 3 years) in diagnosing these types of conditions.

In conclusion, sarcopenia and sarcopenic obesity may be novel risk factors for gastric carcinogenesis. Hence, controlling SMI and BMI as estimators of sarcopenia and sarcopenic obesity may be important for preventing gastric cancer. Our study only identified the association between sarcopenia and sarcopenic obesity and gastric carcinogenesis to prove our hypothesis. Therefore, further research should be performed to clarify the precise mechanism of this association.

## Data Availability Statement

The datasets generated for this study are available on reasonable request to the corresponding author.

## Ethics Statement

The studies involving human participants were reviewed and approved by Institutional Review Board of Gangnam Severance Hospital (IRB no. 3-2018-0349). Written informed consent for participation was not required for this study in accordance with the national legislation and the institutional requirements.

## Author Contributions

YK: substantial contributions to conception and design, analysis and interpretation of data, drafting the article, and revising it critically for important intellectual content. J-HK: substantial contributions to conception and design, final approval of the version to be published, and agreement to be accountable for all aspects of the work. SB: acquisition of data. JC, YY, and HP: revising the article critically for important intellectual content. All authors: review the manuscript.

### Conflict of Interest

The authors declare that the research was conducted in the absence of any commercial or financial relationships that could be construed as a potential conflict of interest.
